# LPInsider: a webserver for lncRNA–protein interaction extraction from the literature

**DOI:** 10.1186/s12859-022-04665-3

**Published:** 2022-04-15

**Authors:** Ying Li, Lizheng Wei, Cankun Wang, Jianing Zhao, Siyu Han, Yu Zhang, Wei Du

**Affiliations:** 1 Key Laboratory of Symbolic Computation and Knowledge Engineering, Ministry of Education, College of Computer Science and Technology, Jilin University, Changchun, 130012 China; 2 Department of Biomedical Informatics, College of Medicine, Ohio State University, Columbus, OH 43210 USA; 3 Department of Computer Science, Faculty of Engineering, University of Bristol, Bristol, BS8 1UB UK

**Keywords:** lncRNA–protein interaction, Corpus, Named entity recognition, Multiple text features, Logistic regression

## Abstract

**Background:**

Long non-coding RNA (LncRNA) plays important roles in physiological and pathological processes. Identifying LncRNA–protein interactions (LPIs) is essential to understand the molecular mechanism and infer the functions of lncRNAs. With the overwhelming size of the biomedical literature, extracting LPIs directly from the biomedical literature is essential, promising and challenging. However, there is no webserver of LPIs relationship extraction from literature.

**Results:**

LPInsider is developed as the first webserver for extracting LPIs from biomedical literature texts based on multiple text features (semantic word vectors, syntactic structure vectors, distance vectors, and part of speech vectors) and logistic regression. LPInsider allows researchers to extract LPIs by uploading PMID, PMCID, PMID List, or biomedical text. A manually filtered and highly reliable LPI corpus is integrated in LPInsider. The performance of LPInsider is optimal by comprehensive experiment on different combinations of different feature and machine learning models.

**Conclusions:**

LPInsider is an efficient analytical tool for LPIs that helps researchers to enhance their comprehension of lncRNAs from text mining, and also saving their time. In addition, LPInsider is freely accessible from http://www.csbg-jlu.info/LPInsider/ with no login requirement. The source code and LPIs corpus can be downloaded from https://github.com/qiufengdiewu/LPInsider.

**Supplementary Information:**

The online version contains supplementary material available at 10.1186/s12859-022-04665-3.

## Background

LncRNA is a type of ncRNA with a length of more than 200 nucleotides, which plays essential roles in various biological processes, such as gene transcription regulation, gene post-transcriptional regulation, epigenetic regulation, and cancer [1–5]. lncRNA–protein interactions (LPIs) is one of the critical mechanisms to conduct multiple essential functions of lncRNAs [6]. Therefore, it is necessary and fundamental to explore LPIs for understanding the molecular mechanism and function of lncRNAs involved in the entire biological system. At present, immunoprecipitation [7], high-throughput sequencing [8], analysis of experimental data based on CLIP-seq [9, 10] and sequence prediction [11] are used to analyze LPIs.

With the exponential growth of biomedical texts, numerous biological entity relation extraction models from the biomedical literature have been widely studied by integrating natural language processing techniques and machine learning models, such as protein–protein interactions (PPIs) [12], drug–drug interactions (DDIs) [13] and chemical–protein interactions [14]. All these different interaction extraction models have corresponding labeled corpora. The reason why there are few text mining models for LPI extraction [15] is that there is no highly reliable labeled corpus of LPI. It is significant to build a computational model to automatically extract LPIs from biomedical texts. Li Ao extracted LPIs by using traditional features and the corpus constructed from PubMed, but it does not provide source code [15]. There is no online webserver for large-scale LPI prediction based on biological literature. We propose a computational model for LPI extraction from biomedical literature.

The naming conventions for biomedical entities can be complicated. For example, the naming conventions for lncRNAs are a complex process. There are nine rules that need to be followed to reasonably name lncRNAs [16]. The three strategies for biomedical named entry recognition (Bio-NER) are rule-based methods [17], dictionary-based methods [18], and machine learning based methods [19]. The rule-based Bio-NER methods separate different classes using a large number of rules, but it does not perform well on larger scale datasets. Dictionary-based Bio-NER methods contain a large collection of names that accurately match entities in the text. In the case of rapidly increasing biomedical texts, this approach is unlikely to uncover emerging categories. Machine learning-based Bio-NER methods utilize statistical-based classification models for named entity recognition, while no longer requiring the researcher to write a large number of rules. [20]. In LPInsider, the dictionary-based named entity identification method is used. However, this method cannot include the latest entity names, so we allow users to upload new entity names for lncRNAs and proteins in our webserver to improving the performance of named entity recognition. The NER model of Stanford CoreNLP uses Conditional Random Fields (CRFs) [21]. We use the tools provided by Stanford CoreNLP [22] to train a named entity recognition method for lncRNAs and proteins. In the Webserver we developed, users can choose one of two methods for named entity recognition.

Text relation extraction can be transformed into text classification. Initially, rule-based methods are used for relationship extraction [23]. This approach requires not only experts but also a lot of detailed rules. For example, rule-based methods may contain rules for prefix and suffix of words and dependency parse trees [24, 25]. To overcome the drawbacks of the rule-based methods, machine learning methods and deep learning methods are used to solve this problem. Relation extraction methods based on machine learning and deep learning are classified as: supervised learning methods [26–28], semi-supervised learning methods [29, 30], and unsupervised learning methods [31–34]. In the LPInsider we developed, after comparing the common deep learning and machine learning based relation extraction methods, we chose a logistic regression classifier [35] using multiple text features for relationship extraction.

The main contributions of LPInsider include: (1) LPInsider for LPIs relation extraction model is constructed by integrating multiple text features (semantic word vectors, syntactic structure vectors, distance vectors and part of speech vector) into logistic regression model. (2) To maximize the feasibility of LPInsider, a webserver with user-friendly interface is developed to free non-experts from programming burden. (3) LPInsider provides a highly reliable LPIs corpus. Positive samples in the corpus are obtained by comparing abstracts on lncRNAs in PubMed with experimentally validated LPIs in LncRInter [36]. Meanwhile, the negative samples in the corpus must match one of the specified two conditions. The corpus of LPI will be a great promotion for ongoing study on LPI extraction. Bioinformatics research has experienced explosive growth in the past decades, demanding higher requirements for permanent data preservation and reproducibility of programs. MIABi [37] represents minimum information about a bioinformatics investigation. LPInsider meets the requirements of MIABi in terms of algorithms, analysis, source code, and webserver.

## Implementation

**Fig. 1:**
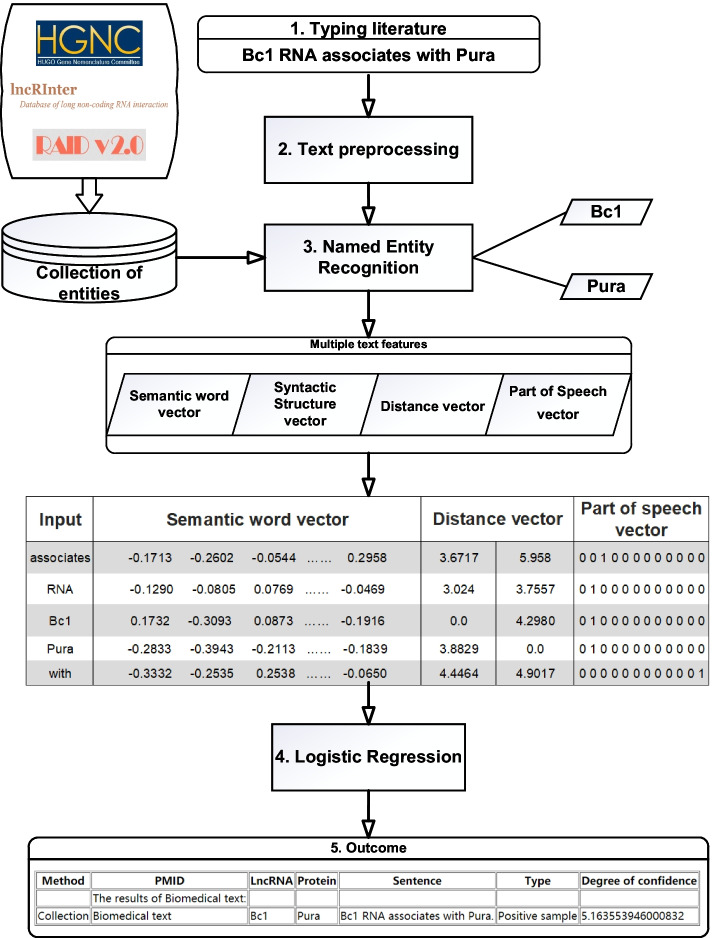
The flowchart of LPInsider. The flowchart consists of six parts: (1) typing literature, (2) text preprocessing, (3) named entity recognition, (4) Extracting text features, (5) classification by logistic regression, (6) outcome including entity information and the judgment of positive or negative samples. For example, after users type “BC1 RNA associates with Pura”, webserver recognizes that BC1 is lncRNA and Pura is protein, and finds the interac-tion between BC1 and Pura. Users get the result returned by webserver

To extract lncRNA–protein interaction, there are four major steps in LPInsider: (1) preprocessing users’ typed literature, (2) named entity recognition, (3) computing multiple text features, (4) building logistic regression model. The flowchart of LPInsider is depicted in Fig. 1.

### Collection of LncRNA and protein library

The names of lncRNAs and proteins are extracted from multiple databases, including RAID v2.0 [38], LncRInter, HGNC [39], GENCODE [40], LncRNADisease [41], UniProt [42], Lnc2Cancer [43], NPInter [44], RPISeq [45] and STRING [46]. Table 1 shows the number of lncRNA and protein in these databases. The names of some entities appear in multiple databases, so the names of this part are only counted once. Finally, there are 92596 names of lncRNAs and 21187257 names of proteins.

**Table 1:** Statistics of lncRNAs and proteins

Database	lncRNAs	Proteins
HGNC	11,513	0
GENCODE	6424	0
LncRNADisease	373	0
Lnc2Cancer	1618	0
RAID v2.0	3460	10,968
LncRInter	277	318
NPInter	76,870	7442
RPISeq	0	2043
UniProt	0	83,692
STRING	0	21,129,733

### Construction of LPIs Corpus

The biomedical literatures in PubMed are retrieved, using the query words, such as ‘long noncoding RNA’, ‘lncRNA’, ‘long non-coding RNA’ and ‘lincRNA’. A total of 18788 abstracts of lncRNA are downloaded. The second step is to break the abstract into sentences. To create highly reliable positive samples, biomedical literatures in PubMed and the experimentally validated and highly reliable LncRInter are used as follows: the sentences containing both lncRNA and protein are found directly in the downloaded abstracts in PubMed, which are subsequently compared with the samples in LncRInter, and select the positive samples.

Negative samples do not exist in LncRInter and match one of the following two conditions: (1) sentences that contain both lncRNA and protein do not contain keywords of interaction, and negative word has no effect on the judgment of negative samples. (2) Sentences contain lncRNA, protein, keywords of interaction, and negative words. Interaction keywords include the verb forms and noun forms of associate, correlate, bind, interact, and enrich. Additional file 1 describes the acquisition of negative samples in more detail. Table 2 hows the comparison of the two types of negative samples of LPInsider. In the table, “True” means the sample contains the item; “False” means the sample does not contain the item; “/” in the first of the two conditions means negative word is included or not included does not affect the judgment of negative sample.

**Table 2:** The comparison of the two types of negative samples

lncRNA	Protein	Interaction keyword	Negative word	Example
True	True	False	/	There was no significant change in Igf2 or H19 expression in brain
True	True	True	True	We found no association between the FISH resultsand MALAT1 expression in patients

The LPIs corpus is constructed manually with 397 high-quality negative samples and 412 high-quality positive samples. Additional file 2 describes the construction of LPIs corpus in detail.

### Typing literature

The webserver allows users multiple ways to enter biomedical text. The users can input PMID or a list of PMID, and then the webserver will automatically download the abstract of the corresponding paper. The user can also enter the PMCID, and then the webserver will automatically download the full text of the corresponding paper. And users can directly input the biomedical text in a specific format. Only English words and common punctuation are allowed. Special characters will be filtered by webserver and will not be included in the result. Additionally, users can upload file containing PMID lists or biomedical texts. This file must be in text format. The PMID list in the file must be separated by the enter key, and cannot contain characters other than numbers.

### Text preprocessing

The abstracts are divided into sentences by tokenizing using nltk toolkit [47]. Stop words and punctuation are removed. Then the remained words in a sentence are lemmatized by the nltk toolkit and are converted into lowercase.

### Named entity recognition

After text processing, the names of LncRNA and protein in the text need to be located, unified and standardized. Stanford CoreNLP [22] allows users to train their own Named Entity Recognition (NER). The corpus of the interaction of lncRNA and protein that we constructed is used to train our NER model. The IntAct [48] dataset is used to verify the precision, recall, and f1-score of the protein identified on our trained NER model, and the LncRNADisease dataset [49] is used to verify the precision, recall, and f1-score of lncRNA identified on our trained NER model. Table 3 shows the results of tenfold cross-validation verification on the corpus we created, IntAct and LncRNADisease. PPInterFinder [50], a tool for extracting causal relations on human proteins from literature, annotates biomedical texts in IntAct and allows researchers to use it. Therefore, we used this annotated IntAct dataset to validate the NER model we created. How to evaluate the NER model using the datasets is explained in detail in Additional file 3.

**Table 3:** The results of tenfold cross-validation verification on LPIs Corpus, IntAct and LncRNADisease

Database	Type	Precision	Recall	f1-score
LPIs Corpus	lncRNA	0.9541	0.9836	0.9686
	protein	0.71857	0.8727	0.7881
LncRNADisease	lncRNA	0.5597	0.5174	0.5378
IntAct	protein	0.4555	0.2300	0.3057

The collection of lncRNAs and proteins is the main method used for NER, and the trained Stanford NER is used as a complementary method to extend the lookup of lncRNA and protein. To facilitate researchers to read and use the program to process the results, two blank lines are added to the query results to distinguish the two different NER methods.

### Extraction of multiple text features

#### Semantic word vector

Semantic word vectors are used to maintain the linear relationship of the words in the sentences. The embedding representations for the majority of words are pretrained from a large-scale text of about 5 billion words found from the titles and abstracts of about 14 million articles in PubMed and the full text of about 700,000 articles in PubMed Central using the word2vec tool contained in NLPLab’s trained word vector model [51], which can be download at http://bio.nlplab.org/. The word2vec tool computes word embedding using the skip-gram model with a window size of 5, hierarchical softmax training, and a frequent word subsampling threshold of 0.001 to create 200-dimensional vectors. The word2vec tool provided by NLPLab is trained from a huge number of biomedical texts in PubMed, but there are still some words in LPI corpus that are not included. Therefore, based on the word2vec tool provided by NLPLab, we use the tool provided by gensim [52] to retrain the word2vec tool specifically for the extraction of LPI can have a more accuracy description of the words.

When users submit biomedical texts, the online webserver prioritizes the use of the retrained word2vec tool. If there are some words in the text that are not included in our trained word2vec tool, then the word2vec tool provided by NLPLab is used to find the word vectors. Finally, if the word2vec tool provided by NLPLab also does not have the word vectors for those words, then zero representation will be used.

#### Syntactic structure vector

The integration of syntactic structure information containing a higher-order syntactic relationship in the sentence into the LPI prediction model can improve the performance of the model. The Stanford Parser of Stanford CoreNLP is used to obtain the syntactic structure of the sentence. The obtained syntactic structure is used to generate the shortest dependency path ordered sequence instead of the original linear order of a sentence. The shortest dependency path ordered sequences as the syntax word vectors are fed into the LPIs prediction model.

#### Distance vector

The next necessary step is to calculate the distance vector between the two entities lncRNA and protein. In a sentence containing both lncRNA and protein entities, we calculate the distance vectors of the three words to the left of the entity and the three words to the right of the entity by calculating the cosine distance between the words and the entity. If the number of words close to the entity is less than three, the distance vector is calculated according to the actual number of words.

For example, in the sentence “Bc1 RNA binds to Eif4a1 with high affinity”, “Eif4a1” is protein, and the three nearest words to the left of “Eif4a1” are “RNA”, “binds” and “to”, while the three words closest to “Eif4a1” on the right are “with”, “high” and “affinity”. We calculate the cosine distances of these six words from “Eif4a1” as distance vectors. The left side of the entity Bc1 has no words and no distance vector is calculated.

#### Part of speech vector

The part of speech corresponding to the word in a sentence is calculated by using the POS_TAG of Stanford CoreNLP. Here 11 types of parts of speech are considered. The one-hot method is used to encode 11 types of parts of speech. The encoding results are shown in Additional file 4. For example, if the part of speech of lncRNA is NN, its encoding for part of speech is [01000000000]. Additional file 5 shows the full form of each abbreviation in Additional file 4. For example, NN is abbreviation for noun.

### LPInsider

The multiple textual features and different machine learning models are integrated to comprehensively conduct feature selection and model evaluation. For each model, the statistical measures, including accuracy, precision, recall, and f1-score are used to evaluate the performance. The classification results of various textual features are compared by tenfold cross-validation on traditional machine learning models (LGBM [53], SVM [54], Logistic Regression [35], Random Forest Classifier [55] and xgboost [56]) and common deep learning models (textCNN [57], LSTM [58] and capsule network [59]).

scikit-learn [60] is a Python module integrating classical machine learning algorithms, providing a variety of Application Programming Interfaces(APIs), including SVM, Logistic Regression and Random Forest Classifier. We use version 0.23.2 of scikit-learn, version 3.2.1 of lightGBM to provide the API for the LGBM classifier and version 1.4.0 of xgboost to provide the API for the classifier. Meanwhile, three common deep learning models are built based on version 1.12.0 of TensorFlow [61] and version 2.2.4 of Keras [62]. In addition, we optimize various hyperparameters for five machine learning models and three deep learning models. Logistic Regression with hyperparameter optimization is the best model.

We introduce separately the parameters of the classifier as LPInsider, and focused on adjusting the hyperparameters “C” and “max_iter” of the logistic regression classifier. These two parameters represent the regularization factor and the maximum number of times the algorithm converges. All other parameters are default parameters.

It should be noted that the TextCNN, LSTM and capsule networks that participated in the comparison are created strictly as in the original article. The network architecture is maintained as in the original article, with changes made only in the data input part. LSTM and TextCNN use binary cross-entropy as the loss function, and capsule networks use a self-defined loss function, while all three models use the Adam optimizer [63]. The machine learning model with the best performance is used to construct our tool LPInsider for LPI prediction.

## Webserver interface and functions

**Fig. 2:**
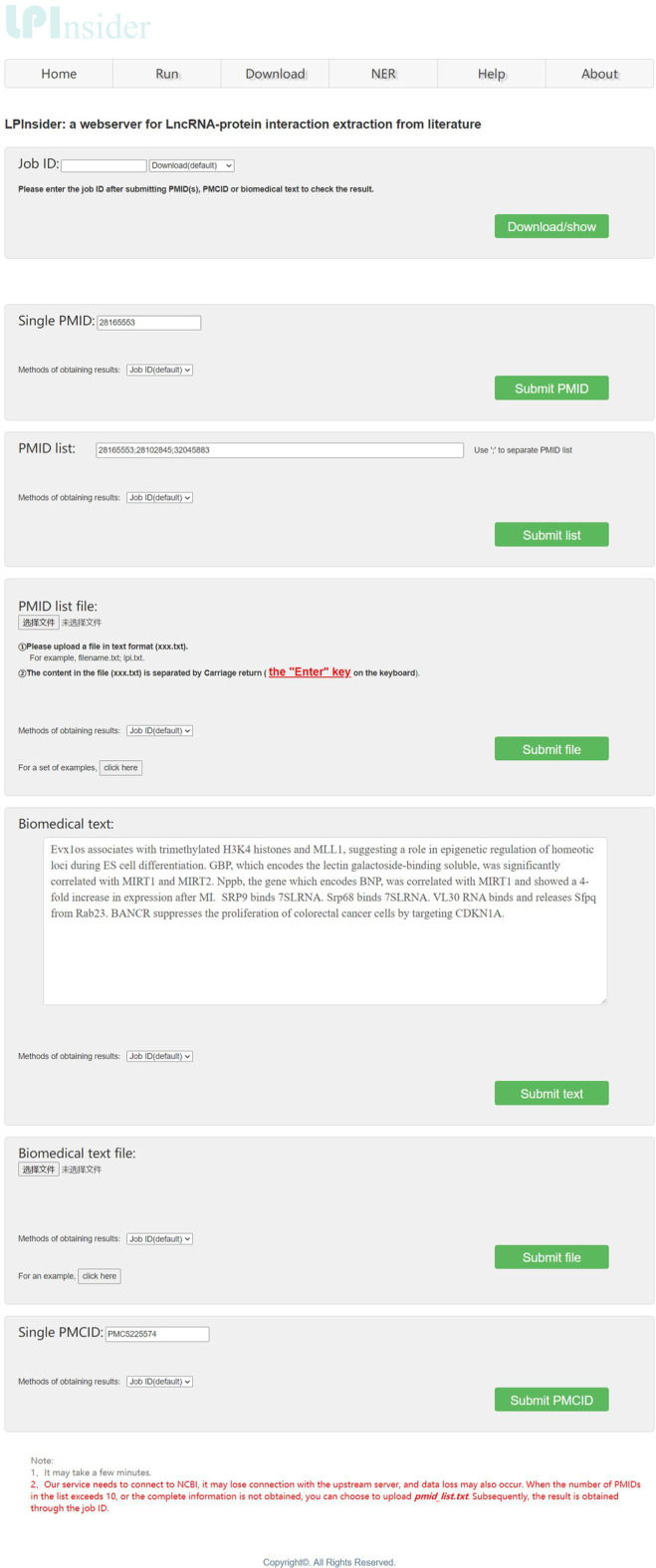
Screenshot of LPInsider’s webserver

**Fig. 3:**
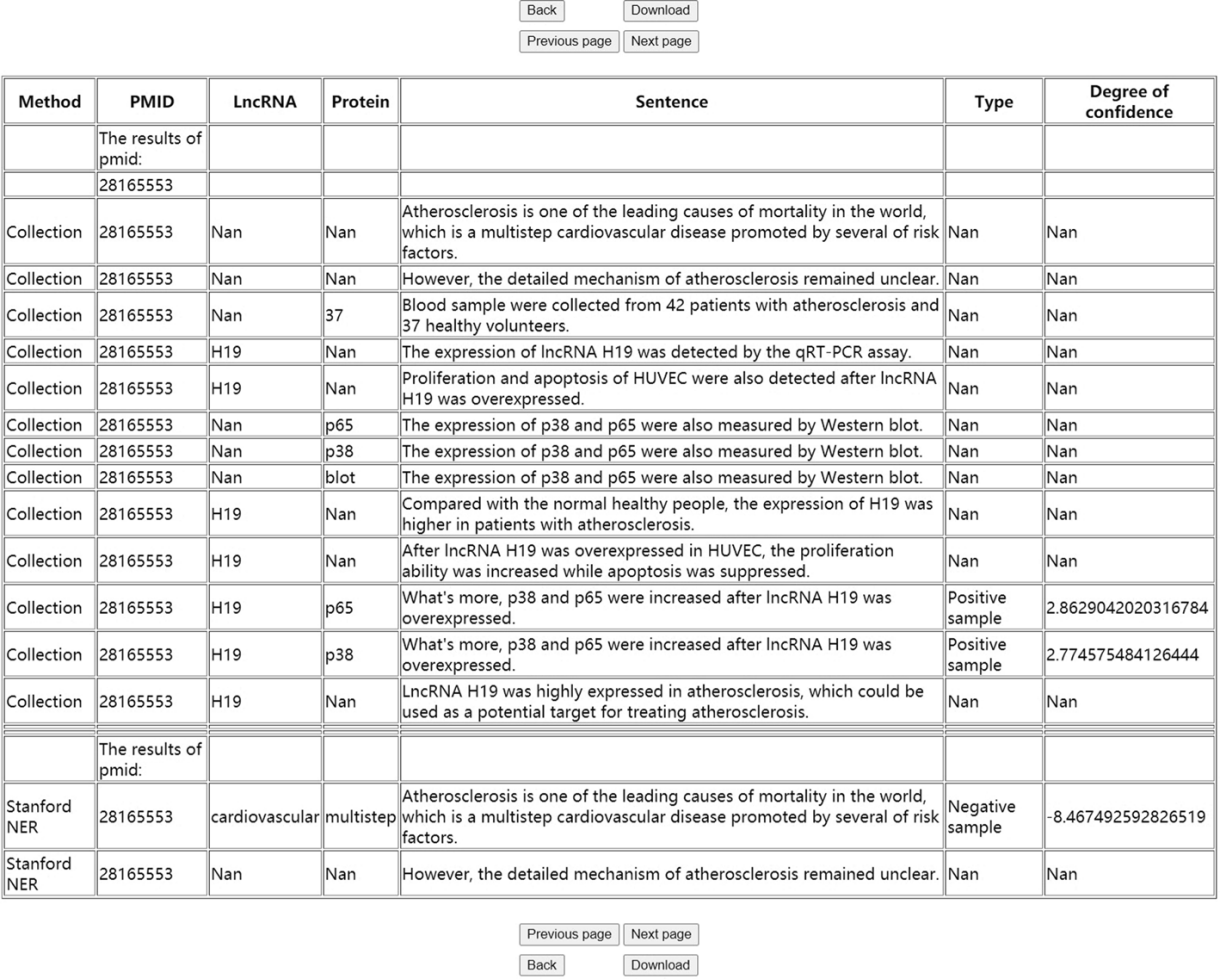
Screenshot of a part of the result returned by webserver

The input of biomedical text and prediction of the results are the main functions of LPInsider’s web server. A screenshot of LPInsider’s webserver is shown in Fig. 2. When PMID, PMCID, PMID List or biomedical text is submitted, users will get a job ID. After waiting for a period of time, users can download or query the results online through this ID. Figure 3 shows a part of the results generated after the user submitted a single PMID of 28165553. The results generated by the webserver have a total of seven columns. The explanation for each column is as follows:

1. The first column is whether the user uses the lncRNA and protein data set or Stanford NER for named entity recognition.
2. The second column shows that the data comes from PMID, PMCID or biomedical text.
3. The third column shows the identified lncRNA.
4. The fourth column shows the identified protein.
5. The fifth column shows the sentences involved in relation extraction.
6. The sixth column is the type of sentences involved in relation extraction. Determine whether it is a positive sample or a negative sample.
7. The seventh column is the confidence of the judgment. In the downloaded file or query results, “Nan” means that this item is empty.

In the downloaded file or query results, “Nan” means that this item is empty.

## Results

**Fig. 4:**
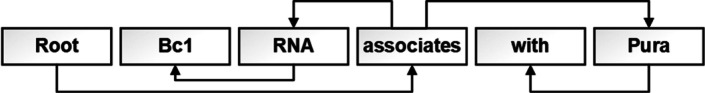
Example of syntactic structure vector

The text of LPIs corpus is converted into a digital representation that a computer can process. Additional file 6 describes an example of only the semantic word vector. The logistic regression classifier works best when only semantic word vectors are used. Figure 4 show an example of syntactic structure vector. The result of using depth-first search (DFS) to traverse “Bc1 RNA associates with Pura” is “associates RNA Bc1 Pura with”. Additional file 7 is an example of describes the data structure using semantic word vectors and syntactic structure vectors. After using 2 types of features, we can find that the logistic regression classifier still works best. Additional file 8 is an example of describes the data structure using semantic word vectors, syntactic structure vectors, and distance vectors. With the addition of the location feature vector, the logistic regression classifier still performs remarkably well. Additional file 9 is an example of semantic word vector, syntactic structure vector, distance vector, and part of speech vector. The performances of five machine learning models have been described in the Table 4. Meanwhile, the model of Logistic Regression is also compared with textCNN, capsule network, and LSTM, and the results are shown in Table 5.

**Table 4:** Using multiple text features

Features	Classifier	Accuracy	Precision	Recall	f1-score
Semantic word vector	LGBM	0.84920	0.86992	0.83037	0.84754
	SVM	0.85534	**0.95127**	0.75734	0.84071
	Logistic regression	**0.88379**	0.91635	**0.85157**	**0.88140**
	Random forest	0.81373	0.84939	0.77344	0.80728
	Xgboost	0.86683	0.87700	0.85637	0.86522
Semantic word vectors and syntactic structure vectors	LGBM	0.85659	0.88094	0.83152	0.85391
	SVM	0.88173	**0.94727**	0.81453	0.87453
	Logistic regression	**0.89657**	0.92684	**0.86788**	**0.89485**
	Random forest	0.82158	0.86687	0.76823	0.81217
	Xgboost	0.87882	0.89386	0.86452	0.87787
Semantic word vectors, syntactic structure vectors and distance vectors	LGBM	0.87476	0.89739	0.85298	0.87276
	SVM	0.89080	**0.95013**	0.83035	0.88486
	Logistic regression	**0.90028**	0.93218	**0.86952**	**0.89838**
	Random forest	0.82360	0.86757	0.77133	0.81454
	Xgboost	0.88382	0.90279	0.86435	0.88169
Semantic wordvector, syntactic structurevector, distance vector and part of speech vector	LGBM	0.89205	0.91488	0.87007	0.89046
	SVM	0.91719	**0.95286**	0.88221	0.91513
	**Logistic regression**	**0.91758**	0.93304	**0.90380**	**0.91722**
	Random forest	0.84216	0.87469	0.80599	0.83753
	Xgboost	0.89164	0.91930	0.86366	0.88926

Bold indicates the better experimental results

**Table 5:** The performances of LPInsider and three deep learning methods

Classifier	Accuracy	Precision	Recall	f1-score
textCNN	0.85935	0.86855	0.85747	0.86022
Capsule network	0.71352	0.71590	0.84718	0.75284
LSTM	0.89497	0.88557	**0.92550**	0.90181
LPInsider	**0.91758**	**0.93304**	0.90380	**0.91722**

Bold indicates the better experimental results

From the statistical experimental results, it is clear that the performance of all five machine learning classifiers improves with the increasing number of features. For example, a logistic regression classifier using four types of features is better than one using three types of features. Similarly, the logistic regression classifier using three types of features is also better than the one using two types of features. Other classifiers also perform better and better as features are added to the model. Four types of features are selected as inputs to the model. It is important to note that the logistic regression classifier performs best among all five machine learning classifiers regardless of which features are fed into the model.

## Conclusion

**Fig. 5:**
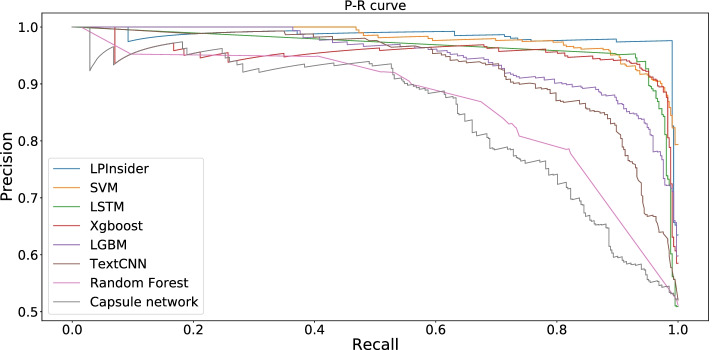
P-R curves of LPInsider with four machine learning and three deep learning models when using multiple text features

LPInsider is an effective webserver for extracting LPIs based on multiple types of text features (semantic word vectors, syntactic structure vectors, distance vectors, and part of speech vectors), and logistic regression. The performance of LPInsider is not inferior to the traditional deep learning algorithm. At the same time, we can also find that the other four machine learning models are not weaker than the three deep learning models being used for classification on our Corpus. Figure 5 shows the P-R curves of LPInsider with multiple machine learning and deep learning models when four text features are used, which proves the above conclusion. Through the cross-validation experiments and comparisons, the optimal textual feature combination including semantic word vectors, syntactic structure vectors, distance vectors and Part of Speech vector consistently achieve the best and most robust performance for the above machine learning models. The performance of logical regression classifier is best. Therefore, the logistic regression classifier trained by LPI Corpus is used for lncRNA–protein interaction extraction.

Although the collection of lncRNA and protein library contains a large number of entity names, some entity names or new names of some entities are not included. Being able to identify the lncRNA and protein entities is crucial for LPIs. So users can submit new lncRNA and protein information to further improve the accuracy of the model [64]. When users submit enough entity names of lncRNAs and proteins, we will use these submitted entity names to retrain our NER model using Stanford CoreNLP, so the ability to identify NER will be improved.

In general, an effective tool for analyzing LPIs, LPInsider, can not only save researchers’ time and reduce resource consumption, but also help researchers to deepen their understanding of lncRNAs through text mining. Additional file 10 introduces a detailed tutorial on using LPInsider webserver. At the same time, a highly reliable corpus of LPI is proposed, which help LPInsider become a valuable text mining tool for ongoing research of LPI.

## Availability and requirements

- Project name: LPInsider
- Project home page: http://www.csbg-jlu.info/LPInsider/
- Operating system(s): Platform independent
- Programming language: Python 3.6.9, Django 2.2.5
- Other requirements: Chrome, Firefox or IE
- License: GNU GPL
- Any restrictions to use by non-academics: None

## Supplementary Information

**Additional file 1.** Acquisition of negative samples.

**Additional file 2.** Construction of LPIs Corpus.

**Additional file 3.** Details of the NER model were evaluated using the datasets IntAct and LncRNA Disease.

**Additional file 4.** Part of Speech vector.

**Additional file 5.** The full form of each abbreviation in Part of Speech.

**Additional file 6.** Example of the semantic word vector.

**Additional file 7.** Example of syntactic structure vector.

**Additional file 8.** Example of syntactic structure vector, and distance vector.

**Additional file 9.** Example of syntactic structure vector, distance vector, and part of speech vector.

**Additional file 10.** Web server description.

## Data Availability

The source code and LPIs corpus can be downloaded from https://github.com/qiufengdiewu/LPInsider.

## Abbreviations

LncRNA: Long non-coding RNA
LPIs: LncRNA–protein interactions
PPIs: Protein–protein interactions
DDIs: Drug–drug interactions
Bio-NER: Bbiomedical named entry recognition
CRFs: Conditional Random Fields
NER: Named Entity Recognition
APIs: Application Programming Interfaces
DFS: Depth-first search
