# Supplementary material for: LPInsider: a webserver for lncRNA–protein interaction extraction from the literature
Source: BMC Bioinformatics. 2022 Apr 15;23:135. doi: 10.1186/s12859-022-04665-3 (PMC9013167; doi:10.1186/s12859-022-04665-3)
Supplement: Supplementary file 1 — Additional file 1. Acquisition of negative samples. [file 12859_2022_4665_MOESM1_ESM.docx]

Additional file 1

**Acquisition of negative samples**

The first step is to download all abstracts about lncRNA by keywords "long noncoding RNA", "lncRNA", "long non-coding RNA" and "lincRNA". A total of 18788 abstracts of lncRNA were obtained. The second step is to break the article into sentences. Finally, artificially select sentences that contain both lncRNA and protein but do not contain keywords of interaction. Interaction keywords include the verb forms and noun forms of associate, correlate, bind, interact, and enrich. The example sentence is "Igf2 and H19 are physically linked imprinted genes." In the example sentence, lncRNA is H19 and protein is Igf2 but there are no keywords for interaction in this sentence. Alternatively, artificially select sentences that contain both lncRNA, protein, keywords of interaction, and negative words. Another example sentence is " NEAT1 has not been shown to bind SR proteins." In this example sentence, lncRNA is NEAT1, protein is SR, and keyword of interaction is bind and the negative word is not. It should be noted that none of the negative samples can appear in LncRInter. If the sentence being filtered exists in LncRInter, then that sentence will not be involved in the filtering of negative samples.
