# Supplementary material for: LPInsider: a webserver for lncRNA–protein interaction extraction from the literature
Source: BMC Bioinformatics. 2022 Apr 15;23:135. doi: 10.1186/s12859-022-04665-3 (PMC9013167; doi:10.1186/s12859-022-04665-3)
Supplement: Supplementary file 2 — Additional file 2. Construction of LPIs Corpus. [file 12859_2022_4665_MOESM2_ESM.docx]

Additional file 2

**Construction of** **LPIs Corpus**

LPInsider integrates the corpus of lncRNA and protein interaction. Firstly, the sentences containing both lncRNA and protein are found directly in the downloaded abstracts in PubMed. In the second step, the sentences are subsequently compared with the samples in LncRInter[[1](#_ENREF_1)], which collects the experimentally verified LPIs from PubMed biomedical literature. The names of lncRNA and protein of the positive sample need to be unified. LncRNA, protein, and sentence in LPInsider's corpus are separated by horizontal tabs. There are 412 positive samples. The downloaded abstracts are split into sentences. Then the sentences in which lncRNA and protein appear simultaneously but do not interact with each other are denoted as negative samples. The total number of negative samples is 397. Users can download LPIs Corpus through the URL (<https://github.com/qiufengdiewu/LPInsider/tree/main/corpus>).

In the positive sample, each line has four items: "lncRNA", "protein", "Interaction Class" and "Description". Similarly in the negative sample, each line contains three items: "lncRNA", "protein" and "Description". The escape character "\t" is used to split the four items in the positive sample and the three items in the negative sample. Examples of LPIs corpus are given in **Table S1.1.** Users can easily use positive and negative samples, and can easily read LPIs corpus using a variety of programming languages. For example, **Table S1.2** gives an example of reading positive and negative samples through Python's package pandas[[2](#_ENREF_2)].

**Table S1.1** Examples of positive and negative samples

| type | lncRNA | protein | interaction class | description |
| --- | --- | --- | --- | --- |
| positive sample | 7SLRNA | SRP9 | RNA-Protein | SRP9 binds 7SLRNA |
|  | Bc1 | Eif4a1 | RNA-Protein | Eif4a1 and PABP interact directly with Bc1 RNA |
| negative sample | H19 | Igf2 | / | There was no significant change in Igf2 or H19 expression in brain |
|  | MALAT1 | FISH | / | We found no association between the FISH results and MALAT1 expression in patients |

**Table S1.2** Example of reading positive and negative samples through Python's package pandas.

| type | code |
| --- | --- |
| positive sample | positive_sample= pandas.read_csv('./pos_sample.txt',sep='\t',header=None) |
| negative sample | positive_sample= pandas.read_csv('./neg_sample.txt',sep='\t',header=None) |

**References**

1. Liu C, Gao C, Ma Z, Cong R, Zhang Q, Guo A: **lncRInter: A database of experimentally validated long non-coding RNA interaction**. *Journal of Genetics and Genomics* 2017, **44**(5):265-268.

2. McKinney W: **pandas: a foundational Python library for data analysis and statistics**. *Python for high performance and scientific computing* 2011, **14**(9):1-9.
