# Supplementary material for: LPInsider: a webserver for lncRNA–protein interaction extraction from the literature
Source: BMC Bioinformatics. 2022 Apr 15;23:135. doi: 10.1186/s12859-022-04665-3 (PMC9013167; doi:10.1186/s12859-022-04665-3)
Supplement: Supplementary file 3 — Additional file 3. Details of the NER model were evaluated using the datasets IntAct and LncRNA Disease. [file 12859_2022_4665_MOESM3_ESM.docx]

Additional file 3

**Details of the NER model were evaluated using the datasets IntAct and LncRNADisease**

PPInterFinder[[1](#_ENREF_1)] is a web-based text mining tool to extract human PPIs from biomedical literature and also annotates biomedical texts in IntAct[[2](#_ENREF_2)]. We present an example of the process of validating our trained NER model with the annotated IntAct dataset. "Seven of the other fifteen clones turned out to be <HumanProtein> ADH1A </HumanProtein>, <HumanProtein> EIF2S3 </ HumanProtein>, NADUF7 , pI esterase , folate <InteractingKeyWord> bind </InteractingKeyWord> protein , arginosuccinate synthetase , and isocitrate dehydrogenase" is a biomedical text in the annotated IntAct provided by PPInterFinder. Firstly, we remove "<HumanProtein>", "</HumanProtein>", "<InteractingKeyWord>" and "</InteractingKeyWord>" from the sentence, and mark "ADH1A" and "EIF2S3" as protein. The lexical properties of the other words were labelled using the tools provided by StanfordCoreNLP[[3](#_ENREF_3)]. Each word and its lexical property form a combination, and the word is separated from their corresponding lexical property by the escape character "\t". Also, the words and their lexical properties are separated by the escape character "\n". Escape characters can be easily processed by computer programs. All sentences in the annotated IntAct undergo similar processing.

The LncRNADisease[[4](#_ENREF_4)] database is not only a resource that curated the experimentally supported lncRNA-disease association data but also a platform that integrated tool(s) for predicting novel lncRNA-disease associations. Similar to using the annotated IntAct dataset to validate the performance of protein recognition on our model, the dataset provided by LncRNADisease was annotated with lncRNA and then annotated with other words using StanfordCoreNLP, also using the escape characters "\t" and "\n" for separation.

Table S1.3 shows examples of the sentence "Seven of the other fifteen clones turned out to be ADH1A, EIF2S3, NADUF7, pI esterase, folate bind protein, arginosuccinate synthetase, and isocitrate dehydrogenase" and the sentence "7SK RNA binding to a HEXIM1 multimer promotes the simultaneous recruitment and hence inactivation of multiple P-TEFb units" after the StanfordCoreNLP annotation process.

**Table S1.3** Example sentences after StanfordCoreNLP annotation processing.

| dataset | sentence | result after processing |
| --- | --- | --- |
| the annotated IntAct | Seven of the other fifteen clones turned out to be ADH1A, EIF2S3, NADUF7, pI esterase, folate bind protein, arginosuccinate synthetase, and isocitrate dehydrogenase | Seven\tCD\nof\tIN\nthe\tDT\nother\tJJ\n  fifteen\tCD\nclones\tNNS\nturned\tVBD\n  out\tRP\nto\tIN\nbe\tVB\n  ADH1A\tprotein\nEIF2S3\tprotein\n  NADUF7\tNN\npI\tNN\nesterase\tNN\n  folate\tNN\nbind\tVBP\nprotein\tNN\n  arginosuccinate\tNN\nsynthetase\tNN\n  and\tCC\nisocitrate\tNN\ndehydrogenase\tNN\n |
| LncRNADisease | 7SK RNA binding to a HEXIM1 multimer promotes the simultaneous recruitment and hence inactivation of multiple P-TEFb units | 7SK\tlncRNA\nRNA\tNN\nbinding\tNN\nto\tTO\n  a\tDT\nHEXIM1\tNN\nmultimer\tNN\n  promotes\tVBZ\nthe\tDT\nsimultaneous\tJJ\n  recruitment\tNN\nand\tCC\nhence\tRB\n  inactivation\tNN\nof\tIN\nmultiple\tJJ\n  PTEFb\tNN\nunits\tNNS\n |

**References**

1. Raja K, Subramani S, Natarajan J: **PPInterFinder—a mining tool for extracting causal relations on human proteins from literature**. *Database* 2013, **2013**.

2. Hermjakob H, Montecchi‐Palazzi L, Lewington C, Mudali S, Kerrien S, Orchard S, Vingron M, Roechert B, Roepstorff P, Valencia A *et al*: **IntAct: an open source molecular interaction database**. *Nucleic Acids Research* 2004, **32**(suppl_1):D452-D455.

3. Manning CD, Surdeanu M, Bauer J, Finkel JR, Bethard S, McClosky D: **The Stanford CoreNLP natural language processing toolkit**. In: *Proceedings of 52nd annual meeting of the association for computational linguistics: system demonstrations: 2014*. 55-60.

4. Geng C, Wang Z, Wang D, Qiu C, Liu M, Xing C, Zhang Q, Yan G, Cui Q: **LncRNADisease: a database for long-non-coding RNA-associated diseases**. *Nucleic Acids Research* 2012(D1):D1.
