# Supplementary material for: LPInsider: a webserver for lncRNA–protein interaction extraction from the literature
Source: BMC Bioinformatics. 2022 Apr 15;23:135. doi: 10.1186/s12859-022-04665-3 (PMC9013167; doi:10.1186/s12859-022-04665-3)
Supplement: Supplementary file 4 — Additional file 4. Part of Speech vector. [file 12859_2022_4665_MOESM4_ESM.docx]

Additional file 4

Part of Speech vector

| Part of speech | One-hot | | | | | | | | | |
| --- | --- | --- | --- | --- | --- | --- | --- | --- | --- | --- |
| JJ | 1 | 0 | 0 | 0 | 0 | 0 | 0 | 0 | 0 | 0 |
| NN | 0 | 1 | 0 | 0 | 0 | 0 | 0 | 0 | 0 | 0 |
| VB | 0 | 0 | 1 | 0 | 0 | 0 | 0 | 0 | 0 | 0 |
| RB | 0 | 0 | 0 | 1 | 0 | 0 | 0 | 0 | 0 | 0 |
| CC | 0 | 0 | 0 | 0 | 1 | 0 | 0 | 0 | 0 | 0 |
| IN | 0 | 0 | 0 | 0 | 0 | 1 | 0 | 0 | 0 | 0 |
| CD | 0 | 0 | 0 | 0 | 0 | 0 | 1 | 0 | 0 | 0 |
| MD | 0 | 0 | 0 | 0 | 0 | 0 | 0 | 1 | 0 | 0 |
| PRP | 0 | 0 | 0 | 0 | 0 | 0 | 0 | 0 | 1 | 0 |
| WDT | 0 | 0 | 0 | 0 | 0 | 0 | 0 | 0 | 0 | 1 |
