# Supplementary material for: LPInsider: a webserver for lncRNA–protein interaction extraction from the literature
Source: BMC Bioinformatics. 2022 Apr 15;23:135. doi: 10.1186/s12859-022-04665-3 (PMC9013167; doi:10.1186/s12859-022-04665-3)
Supplement: Supplementary file 5 — Additional file 5. The full form of each abbreviation in Part of Speech. [file 12859_2022_4665_MOESM5_ESM.docx]

Additional file 5

The full form of each abbreviation in Part of Speech

| abbreviation | full form |
| --- | --- |
| JJ | adjective |
| NN | noun |
| VB | verb |
| RB | adverb |
| CC | conjunction |
| IN | preposition |
| CD | numeral |
| MD | modal auxiliary |
| PRP | pronoun, personal |
| WDT | WH-determiner |
| OTH | Others |
