# Supplementary material for: LPInsider: a webserver for lncRNA–protein interaction extraction from the literature
Source: BMC Bioinformatics. 2022 Apr 15;23:135. doi: 10.1186/s12859-022-04665-3 (PMC9013167; doi:10.1186/s12859-022-04665-3)
Supplement: Supplementary file 7 — Additional file 7. Example of syntactic structure vector. [file 12859_2022_4665_MOESM7_ESM.docx]

Additional file 1

Example of syntactic structure vector

| input | semantic word vector |
| --- | --- |
| associates | -0.1713 -0.2602 -0.0544 …… 0.2958 |
| RNA | -0.1290 -0.0805 0.0769 …… -0.0469 |
| Bc1 | 0.1732 -0.3093 0.0873 …… -0.1916 |
| Pura | -0.2833 -0.3943 -0.2113 …… -0.1839 |
| with | -0.3332 -0.2535 0.2538 …… -0.0650 |
