# Supplementary material for: LPInsider: a webserver for lncRNA–protein interaction extraction from the literature
Source: BMC Bioinformatics. 2022 Apr 15;23:135. doi: 10.1186/s12859-022-04665-3 (PMC9013167; doi:10.1186/s12859-022-04665-3)

Additional file 10

**Web Server Description**

**Introduction**

This webserver is developed on Python, Django. The webserver contains two main functions:

1. The server allows users to extract LPI in five ways. These five methods include PMID, PMID list, text file containing PMID list, biomedical text and PMCID. Users can then get the result by the job ID.
2. The server allows users to upload the entity information of lncRNA and protein to help improve the accuracy of named entity recognition (NER).

The web server is available at <http://www.csbg-jlu.info/LPInsider/>. The web server has been tested on several different browsers, including Microsoft Internet Explorer, Mozilla Firefox and Google Chrome.

This additional file provides a brief instruction to display the usage of the web server of LPInsider.


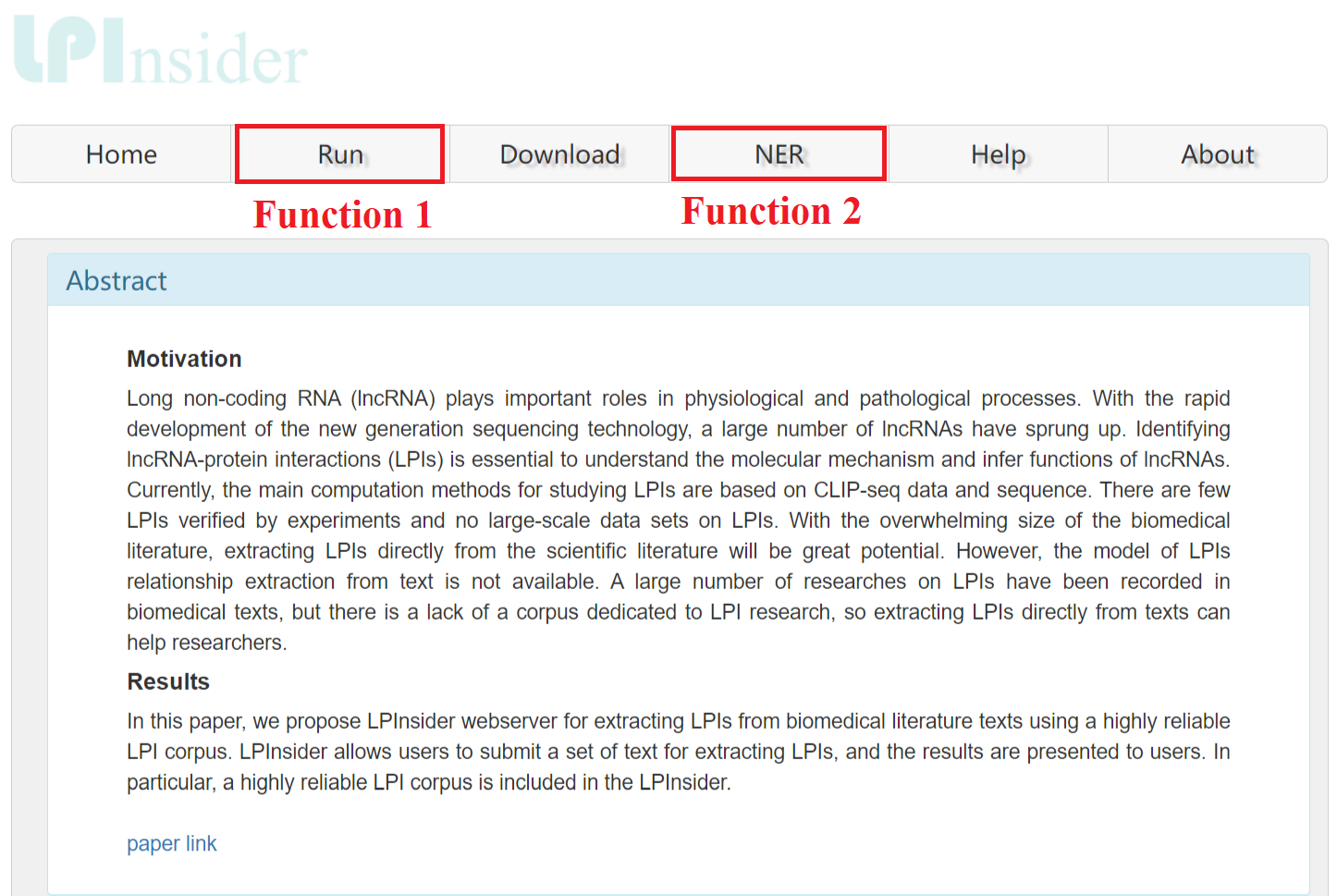


**lncRNA-protein interaction extraction**

LncRNA-protein interaction (LPI) extraction is the main work of the server. The server allows users to extract LPI in multiple Methods: PMID, PMID list, text file containing PMID list or biomedical text, biomedical text and PMCID.

Users need to do the following work:

First, click on the “Run” button to go to the extraction function page. Second, select one of six methods to extract LPI. Third, download or show the results through the job ID returned by the server. Or, when submitting a PMID(s), PMCID or text, the user can choose to receive the results by using the email. The server may take several minutes or hours to process the data, so please check the results later. At the same time, downloading results through job ID is the default method, and users can also choose to show results online.


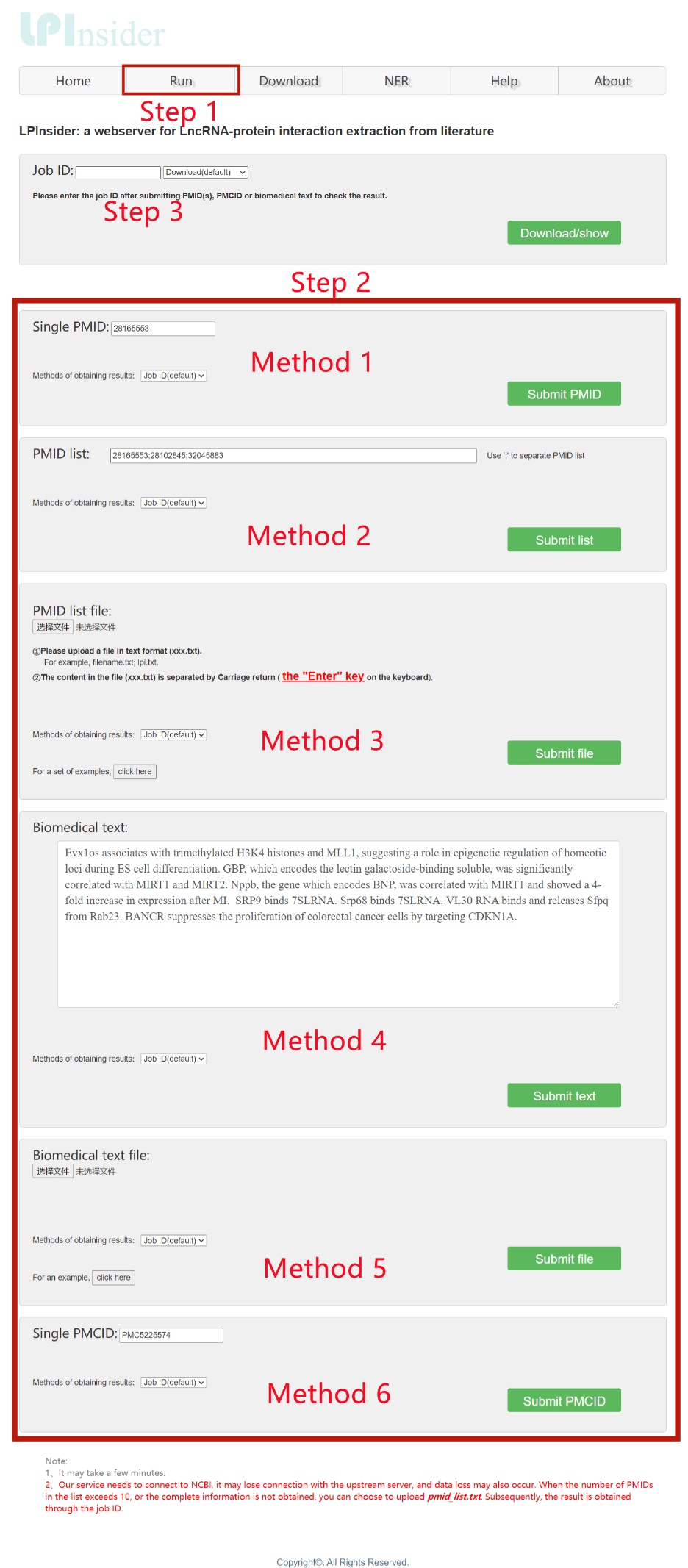


**NER**

Some lncRNAs or proteins are not included in our collection, so users can upload their known entity names to help improve the accuracy of NER. After manual verification, the names will be included in the collection.

Users need to do the following work:

First, click “NER” to enter the upload page. Second, choose whether the entity belongs to lncRNA or protein. Third, enter the name of the entity. Fourth, input the explanation of the entity to help us understand the entity. Fifth, click "Upload" to upload the input information.


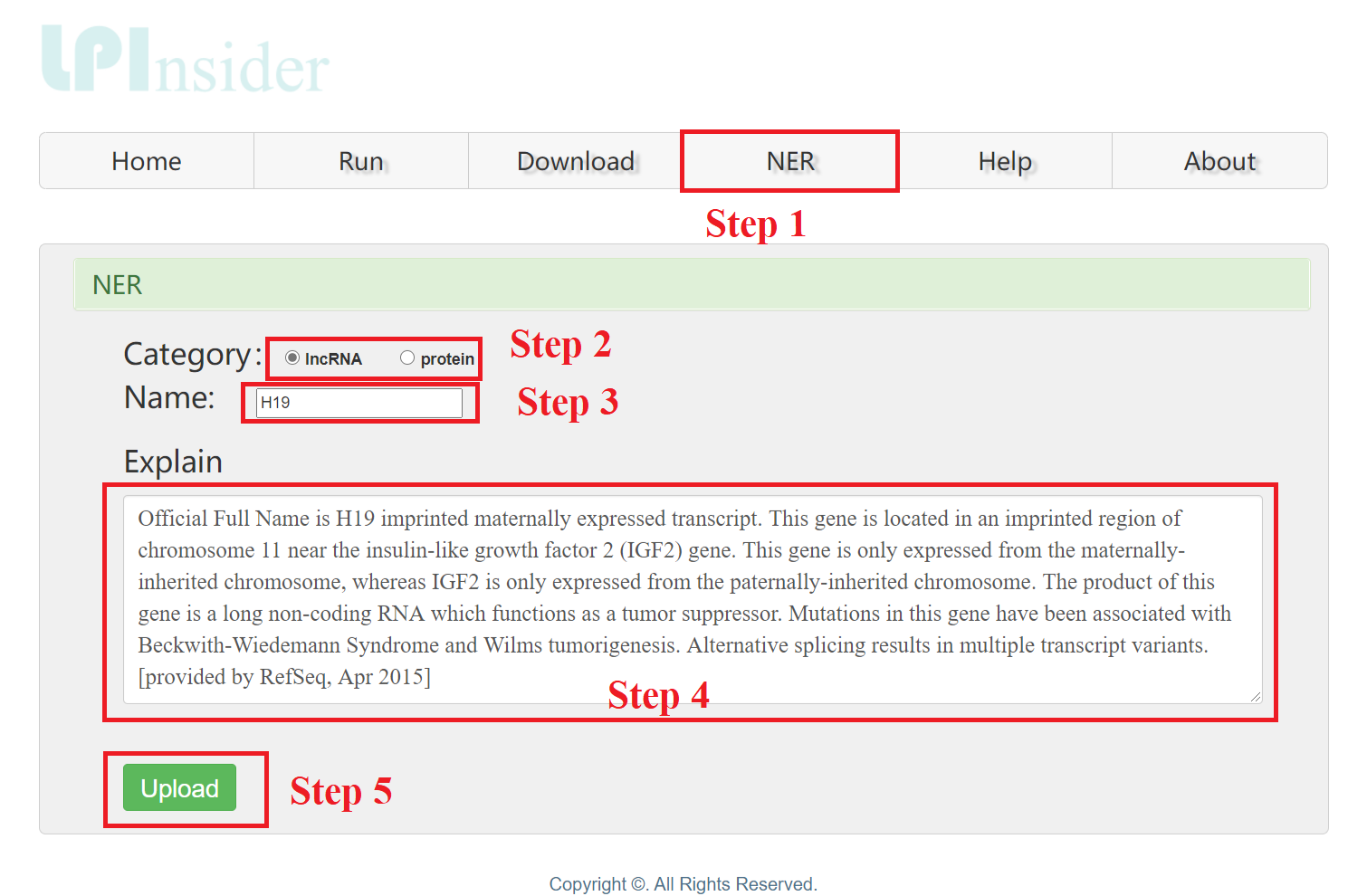

Supplement: Supplementary file 10 — Additional file 10. Web server description. [file 12859_2022_4665_MOESM10_ESM.docx]
